# Hemodynamic characteristics of hyperplastic remodeling lesions in cerebral aneurysms

**DOI:** 10.1371/journal.pone.0191287

**Published:** 2018-01-16

**Authors:** Kazuhiro Furukawa, Fujimaro Ishida, Masanori Tsuji, Yoichi Miura, Tomoyuki Kishimoto, Masato Shiba, Hiroshi Tanemura, Yasuyuki Umeda, Takanori Sano, Ryuta Yasuda, Shinichi Shimosaka, Hidenori Suzuki

**Affiliations:** 1 Department of Neurosurgery, Mie University Graduate School of Medicine, Tsu, Mie, Japan; 2 Department of Neurosurgery, Mie Chuo Medical Center, National Hospital Organization, Tsu, Mie, Japan; 3 Department of Neurosurgery, Suzuka Kaisei Hospital, Suzuka, Mie, Japan; 4 Department of Neurosurgery, Ise Red Cross Hospital, Ise, Mie, Japan; Universitatsklinikum Freiburg, GERMANY

## Abstract

**Background & purpose:**

Hyperplastic remodeling (HR) lesions are sometimes found on cerebral aneurysm walls. Atherosclerosis is the results of HR, which may cause an adverse effect on surgical treatment for cerebral aneurysms. Previous studies have demonstrated that atherosclerotic changes had a correlation with certain hemodynamic characteristics. Therefore, we investigated local hemodynamic characteristics of HR lesions of cerebral aneurysms using computational fluid dynamics (CFD).

**Methods:**

Twenty-four cerebral aneurysms were investigated using CFD and intraoperative video recordings. HR lesions and red walls were confirmed on the intraoperative images, and the qualification points were determined on the center of the HR lesions and the red walls. The qualification points were set on the virtual operative images for evaluation of wall shear stress (WSS), normalized WSS (NWSS), oscillatory shear index (OSI), relative residence time (RRT), and aneurysm formation indicator (AFI). These hemodynamic parameters at the qualification points were compared between HR lesions and red walls.

**Results:**

HR lesions had lower NWSS, lower AFI, higher OSI and prolonged RRT compared with red walls. From analysis of the receiver-operating characteristic curve for hemodynamic parameters, OSI was the most optimal hemodynamic parameter to predict HR lesions (area under the curve, 0.745; 95% confidence interval, 0.603–0.887; cutoff value, 0.00917; sensitivity, 0.643; specificity, 0.893; P<0.01). With multivariate logistic regression analyses using stepwise method, NWSS was significantly associated with the HR lesions.

**Conclusions:**

Although low NWSS was independently associated with HR lesions, OSI is the most valuable hemodynamic parameter to distinguish HR lesions from red walls.

## Introduction

Recent advancement of computational fluid dynamics (CFD) has brought the novel understanding of hemodynamics such as rupture status,[1] rupture point,[2] and hemostatic pattern of cerebral aneurysms.[3]

Atherosclerotic lesions are observed at aneurysmal dome, the parent artery and branches during microsurgery, and these thick and rigid walls may have unfavorable effects on aneurysm clipping or temporary clipping of the parent artery. Atherosclerosis is thought to be the results of hyperplastic remodeling (HR), which may reflect a compensatory hemodynamic reaction for cerebral aneurysms. Previous reports demonstrated that atherosclerosis had a correlation with low wall shear stress (WSS), high oscillatory shear index (OSI), and prolonged relative residence time (RRT) in aorta and carotid artery walls.[4][5][6][7][8] A recent study showed that an area with prolonged RRT had atherosclerosis on cerebral aneurysm walls.[9] Atherosclerosis of aneurysm dome and cerebral arteries were also consistent with an area of high OSI.[10] OSI was formulated to account for the cyclic departure of the WSS vector from its predominant axial alignment.[9] Aneurysm formation indicator (AFI) is a hemodynamic parameter which could quantify the directional change of WSS for detecting the flow stagnation zone.[11] Although the definition is different from OSI, AFI could also measure the fluctuation of WSS vector as well as OSI.[11] Therefore, we supposed that AFI would also be related with HR lesions. In this study, we investigated the relationships between local hemodynamics and HR lesions to determine the most valuable hemodynamic parameters in predicting HR lesions in cerebral aneurysms.

## Materials and methods

This study was conducted in accordance with the guideline and under the approval of the ethics review board of Mie Chuo Medical Center. The requirement for informed consent was waived. Patient records and geometric data were anonymized prior to analysis. We reviewed our database for the following cerebral aneurysms treated at Mie Chuo Medical Center between 2008 and 2017. The inclusion criteria were: 1) saccular aneurysms treated by direct surgery; 2) those that were evaluated by both subtraction three-dimensional (3D) computed tomographic angiography (CTA) using 64-detector multi-slice computed tomography (CT) and CFD before the surgical treatment; and 3) those that had either HR lesions or red walls, or both on the dome detected by microsurgical observation. Cases in which aneurysm wall could not be observed sufficiently in intraoperative images were excluded. Consequently, 24 aneurysms (24 patients) were included in this study (10 ruptured and 14 unruptured aneurysms). There were 13 aneurysms in the middle cerebral artery (MCA), 9 aneurysms in the internal carotid artery (ICA), and 2 aneurysms in the anterior cerebral artery (ACA). Thirteen aneurysms had both HR lesions and red walls, 6 aneurysms had only HR lesions, and 5 aneurysms had only red walls (Table 1).

**Table 1:** Clinical characteristics of cerebral aneurysms.

Case No.	Aneurysm Location	Rupture Status	HR lesions, n	Red walls, n
1	MCA	Unruptured	4	1
2	ICAW	Unruptured	1	1
3	MCA	Ruptured	1	1
4	ICPC	Ruptured	1	1
5	MCA	Unruptured	2	0
6	ICPC	Ruptured	2	1
7	MCA	Unruptured	1	1
8	ICPC	Unruptured	0	1
9	MCA	Unruptured	1	0
10	ACA	Unruptured	3	2
11	MCA	Ruptured	1	2
12	ICAC	Unruptured	2	1
13	ICPC	Ruptured	0	1
14	ICPC	Ruptured	0	1
15	MCA	Unruptured	1	4
16	ICA	Unruptured	0	1
17	MCA	Ruptured	2	0
18*	ICA	Unruptured	2	0
19	MCA	Unruptured	1	3
20	MCA	Ruptured	1	0
21	MCA	Ruptured	0	1
22	MCA	Unruptured	2	2
23	ACA	Ruptured	1	0
24	MCA	Unruptured	1	3

ACA indicates anterior cerebral artery; HR, hyperplastic remodeling; ICAC, internal carotid artery-anterior choroidal artery bifurcation; ICAW, anterior wall of the internal carotid artery; ICPC, internal carotid artery-posterior communicating artery bifurcation; and MCA, middle cerebral artery.

*Case No. 18 was excluded from analyses because of the calculation error.

**Fig 1:**
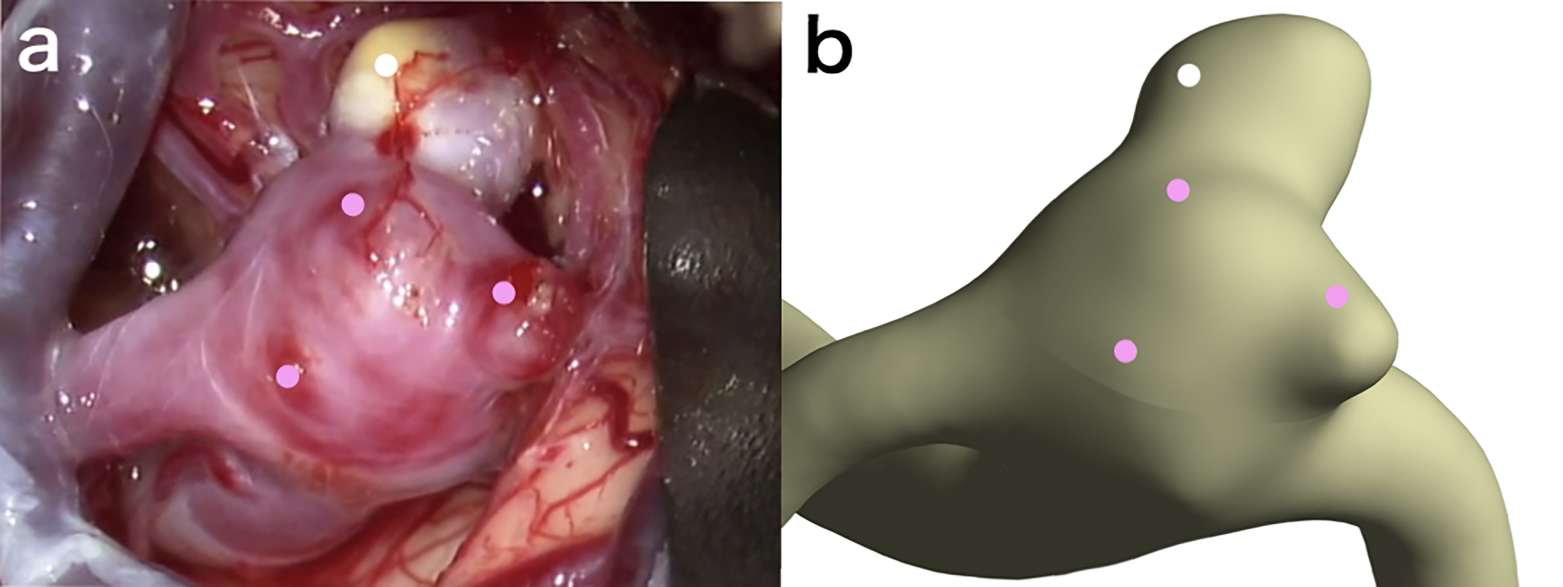
Comparison of intraoperative image (a) and virtual operative image of the same angle as the intraoperative image (b). The qualification points are set on both images as white or pink dots. White dots are set on the hyperplastic remodeling lesion, and pink dots on the red walls.

**Fig 2:**
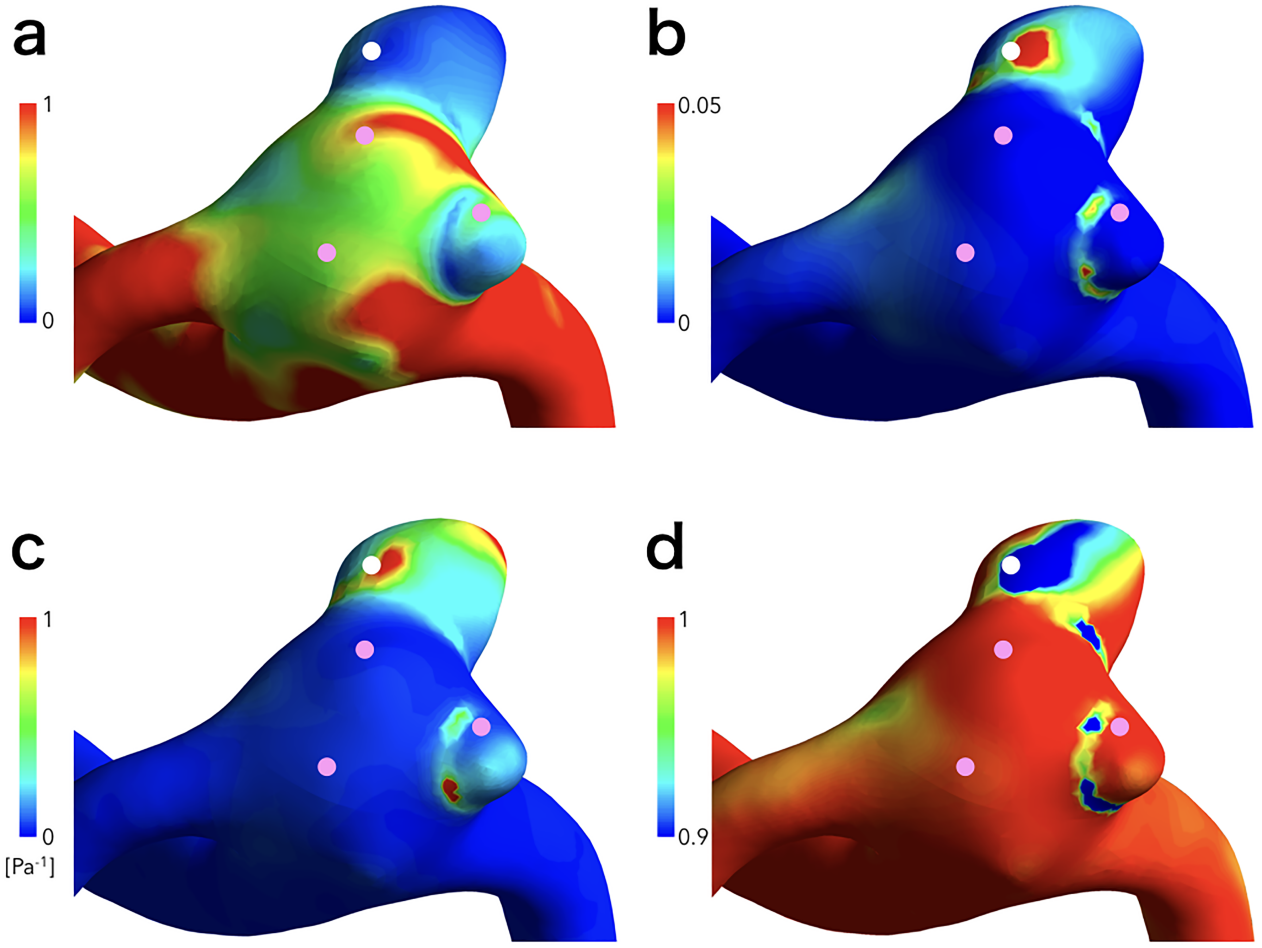
Virtual operative images of normalized wall shear stress (a), oscillatory shear index (b), relative residence time (c), and aneurysm formation indicator (d), with the qualification points.

HR lesions and red walls of aneurysms were assessed on intraoperative images, and qualification points were determined on the center of the HR lesions and the red walls. Evaluation of intraoperative images was performed by two independent experienced neurosurgeons, who judged “obviously” white-colored aneurysm wall to be a HR lesion and “obviously” red-colored aneurysm wall to be a red wall: HR lesions and red walls were adopted when judgment by each evaluator was consistent. As a result, 7 of 65 selected lesions were excluded in this study. In addition, virtual operative geometry images in the same direction as the intraoperative images were prepared and the qualification points were set at the center of the lesions on the virtual operative image by consensus of the 2 independent neurosurgeons blinded to the results of CFD analyses (Fig 1). Then, 2 CFD analysts evaluated hemodynamic parameters at the qualification points with agreement. The hemodynamic parameters including WSS, normalized WSS (NWSS), OSI, RRT, and AFI were compared between HR lesions and red walls (Fig 2).

### Numerical simulations

#### CFD analysis

The patient-specific geometries were generated as stereolithography (STL) with the preoperative 3D-CTA using a Toshiba Aquilion 64 (Toshiba Medical System, Otawara, Japan). Arterial lumen was segmented based on the intra-arterial CT value. The threshold CT value was determined at 40% between the maximum value in the extracranial ICA and the minimum values at the periarterial area by the analytical software (Mimics Innovation Suite; Matelialise Japan, Yokohama, Japan). The STL was remeshed to improve the quality of the surface triangles.

The computational hybrid meshes were generated with tetrahedral and prism elements (ANSYS ICEM CFD17.2; ANSYS, Inc., Canonsburg, PA, USA). Tetrahedral element sizes ranged from 0.1 to 0.6 mm. Six prismatic boundary layers with a total thickness of 0.15 mm covered the vessel wall to locally ensure an accurate definition of the velocity gradient. A straight inlet extension was added to the inlet section to obtain fully developed laminar flow. For the fluid domain, 3D incompressible laminar flow fields were obtained by solving the continuity and Navier-Stokes equations. Numeral modeling was performed using a commercially available CFD package (ANSYS CFX CFD17.2; ANSYS, Inc., Canonsburg, PA, USA). Blood was assumed to be an incompressible Newtonian fluid with a blood density of 1056 kg/m^3^ and a blood dynamics viscosity of 0.0035 Pa · s. Typical flow waveform of phase-contrast magnetic resonance imaging was scaled to achieve a physiological WSS. Traction-free boundary conditions were applied at the outlets. Transient analysis was performed after initial value specification using the results of steady state analysis. The time steps were 0.0001 second, and one pulsatile cycle was taken as output.

#### Hemodynamic parameters calculation

We calculated five hemodynamic parameters including WSS, NWSS, OSI, RRT, and AFI as previously reported [1][2][5][6][11], as follows:

*WSS* indicates the frictional force exerted by the flowing blood tangentially on the dome wall during the cardiac cycle. We defined time-averaged WSS as *WSS*, given by the following equation:

$$WSS=\frac{1}{T}\int_{0}^{T}|{WSS}_{i}|dt$$

where *WSS*_*i*_ is the instantaneous shear stress vector and *T* is the duration of the cycle.

*NWSS* was defined as the WSS ratio to the WSS magnitude of the parent artery. WSS distributions were normalized to the average parent vessel WSS in the same patient to allow comparison of WSS magnitude among different patients.[1] The C1 and C2 segments of ICA were defined as the parent artery of ICA aneurysms; A1 segment as that of ACA aneurysms; and M1 segment as that of MCA aneurysms.

*OSI* was defined as the directional changes of WSS vector during the cardiac cycle, given by the following equation:

$$OSI=\frac{1}{2}\{1-\frac{\left|\int_{0}^{T}{WSS}_{i}dt\right|}{\int_{0}^{T}|{WSS}_{i}|dt}\}$$

*RRT* is an indicator of low or oscillatory WSS reflecting the residence time of blood near the wall, inversely proportional to the magnitude of the WSS vector, given by the following equation:

$$RRT=\frac{1}{\frac{1}{T}|\int_{0}^{T}{WSS}_{i}dt|}$$

*AFI* was defined as the cosine of the angle between *WSS*_*i*_ and *WSS* at the midsystolic deceleration, given by the following equation [11]:

$$AFI=cos(\theta )=\frac{{WSS}_{i}\cdot WSS}{\left|{WSS}_{i}|*|WSS\right|}$$

### Statistical analysis

As not all hemodynamic parameters were normally distributed, Mann-Whitney U test was used to compare each hemodynamic parameter between HR lesions and red walls. Post hoc power analysis was performed to evaluate whether our data had sufficient verification power, and ≥0.8 of power was considered to criteria for validation. The receiver-operating characteristic (ROC) curve analysis was performed for significant hemodynamic parameters on univariate analyses. Intercorrelations between hemodynamic parameters were examined using Spearman’s rank correlation test, and multivariate logistic regression analyses were performed to clarify independent hemodynamic factors related to HR lesions after excluding parameters with multiple collinearity. Two-sided P values of 0.05 or less were considered to indicate statistical significance for all statistical tests.

All statistical analyses were performed with EZR (Saitama Medical Center, Jichi Medical University, Saitama, Japan), which is a graphical user interface for R (The R Foundation for Statistical Computing, Vienna, Austria).

## Results

**Fig 3:**
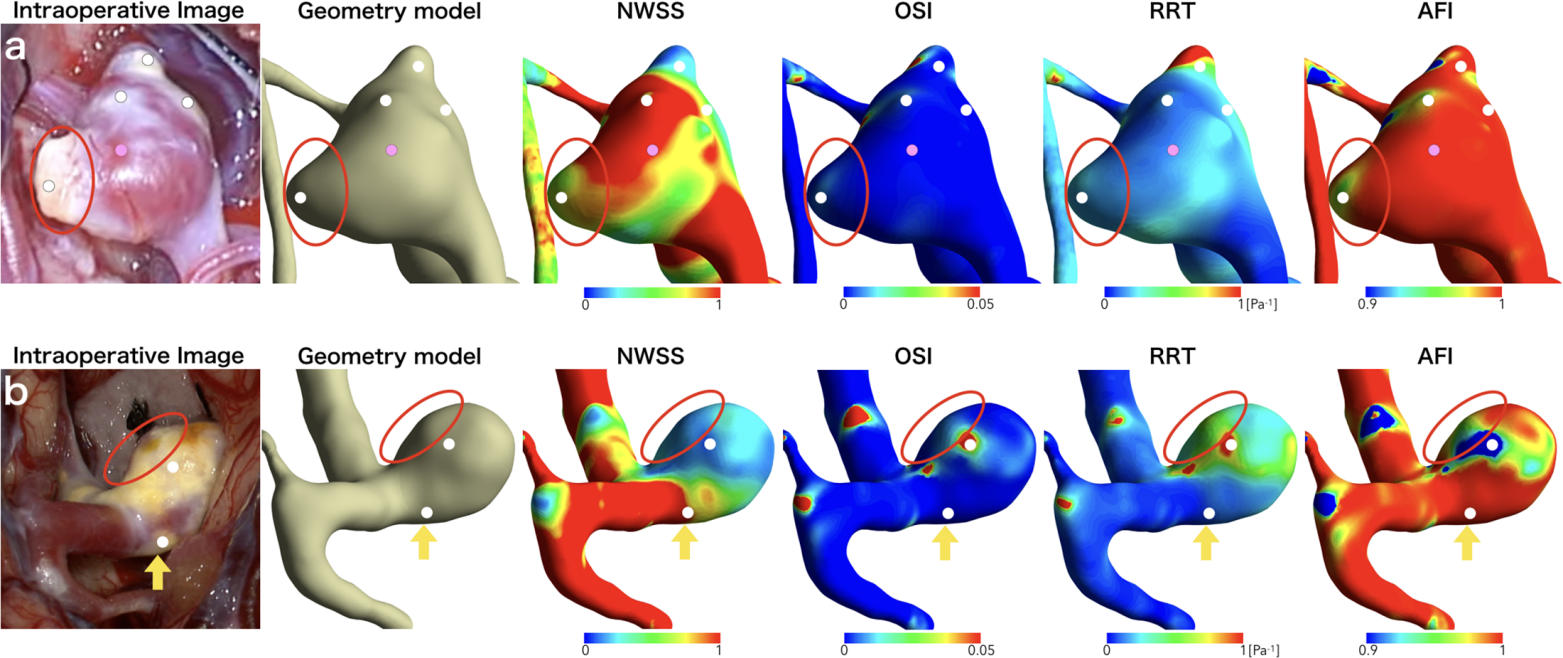
Comparison of intraoperative image and visualized hemodynamic parameters in 2 cases: middle cerebral artery aneurysm (a) and internal carotid artery-posterior communicating artery bifurcation aneurysm (b). The shape of aneurysm in the intraoperative image is partly different from that in geometry model because of the atherosclerotic hyperplastic remodeling of the wall (red circle). The qualification points are set on images as white (indicating hyperplastic remodeling lesion) or pink (indicating red wall) dots. Low normalized wall shear stress (NWSS), high oscillatory shear index (OSI), low aneurysm formation indicator (AFI), and prolonged relative residence time (RRT) are observed at or near most of hyperplastic remodeling lesions, but one hyperplastic remodeling lesion is located on the area with high NWSS, low OSI, high AFI, and shortened RRT (yellow arrow).

Thirty-five HR lesions and 30 red walls were identified from 24 cases. Five HR lesions were excluded because 4 lesions were considered as continuous lesions from another one and one lesion as reflection by the light of operating microscope. In addition, 2 red walls were excluded because they were suspected as hematoma. Therefore, 30 HR lesions and 28 red walls were adopted in this study. Variations in hemodynamic parameters including low NWSS, high OSI, low AFI, and prolonged RRT were observed at or near HR lesions (Figs 1, 2 and 3), but a certain trend was not shown at or near red walls.

On statistical evaluations, an ICA aneurysm with two HR lesions was excluded because the hemodynamic parameters could not be calculated due to calculation errors. A MCA aneurysm with two HR lesions was also not added to statistical evaluation of AFI because there was a calculation error of AFI. Therefore, hemodynamic parameters except for AFI were compared statistically between 28 HR lesions and 28 red walls, and only AFI was compared between 26 HR lesions and 28 red walls (Table 1). HR lesions had significantly lower NWSS, higher OSI, higher RRT, and lower AFI than red walls had (0.119 versus 0.475, P = 0.00822; 0.015 versus 0.0020, P = 0.0136; 1.07 /Pa versus 0.225 /Pa, P = 0.036; and 0.973 versus 0.997, P = 0.0419, respectively), but there was no significant difference in WSS (1.079 versus 3.709, P = 0.0537) (Table 2). As a result of post hoc power analysis, OSI and NWSS had adequate power (0.938 and 0.884, respectively), but other hemodynamic parameters did not have enough power (WSS, 0.267; RRT, 0.0397; and AFI, 0.533).

**Table 2:** Summary of hemodynamic parameters in hyperplastic remodeling (HR) lesions and red walls.

Parameter	HR lesion (n = 28)	Red Wall (n = 28)	P Value*
WSS, Pa	1.08 (0.270–6.43)	3.71 (1.58–7.36)	0.054
NWSS	0.119 (0.0608–0.484)	0.475 (0.248–1.07)	0.008
OSI	0.0152 (0.00456–0.0876)	0.00158 (0.000540–0.00533)	0.001
RRT, Pa^-1^	1.07 (0.150–5.18)	0.255 (0.130–0.816)	0.036
AFI	0.973 (0.570–0.998)**	0.997 (0.989–0.999)	0.042

AFI indicates aneurysm formation indicator; NWSS, normalized WSS; OSI, oscillatory shear index; RRT, relative residence time; and WSS, wall shear stress. Values are the median (interquartile range).

*Mann–Whitney U test.

**n = 26 in AFI of HR lesions.

The area under the curve (AUC), cut-off point, sensitivity, and specificity of each parameter by analysis of the ROC curve were shown in Table 3. OSI was the most adequate hemodynamic parameter to discriminate HR lesions from red walls (AUC, 0.745; 95% confidence interval, 0.603–0.887; cutoff value, 0.00917; sensitivity, 0.643; specificity, 0.893; P<0.01).

**Table 3:** Receiver operating characteristic (ROC) curve analysis for 4 hemodynamic parameters.

Parameter	AUC	95%CI	Sensitivity	Specificity	Cut-off Value
NWSS	0.704	0.564–0.844	0.571	0.786	0.192
OSI	0.745	0.603–0.887	0.643	0.893	0.00917
RRT, Pa^-1^	0.663	0.515–0.811	0.571	0.821	0.879
AFI	0.662	0.506–0.818	0.654	0.714	0.993

AFI indicates aneurysm formation indicator; AUC, area under the ROC curve; CI, confidence interval; NWSS, normalized wall shear stress; OSI, oscillatory shear index; and RRT, relative residence time.

There were very high correlations between WSS and RRT (correlation coefficient: R = -0.994, P<0.0001; coefficient of determination: R^2^ = 0.988), AFI and OSI (R = -0.931, P<0.0001; R^2^ = 0.867), high correlations between WSS and NWSS (R = 0.8022, P<0.0001; R^2^ = 0.830), moderate correlations between WSS and OSI (R = -0.622, P<0.0001; R^2^ = 0.387), and OSI and RRT (R = -0.652, P<0.0001; R^2^ = 0.425). With multivariate logistic regression analyses using stepwise method (WSS and AFI were excluded because of multiple collinearity), NWSS was significantly associated with the HR lesions (odds ratio, 4.810; 95% confidence interval, 1.370–16.90; P = 0.0141) (Table 4).

**Table 4:** Logistic regression analysis of independent parameters associated with hyperplastic remodeling lesions of aneurysms, with stepwise regression using P values to drop variables.

Step 1			
Parameter	OR	95% CI	P-Value
NWSS	3.24	0.791–13.3	0.102
OSI	5.46E-05	1.67E-11–178	0.200
RRT	1.00	1.00–1.00	0.781
Step 2			
Parameter	OR	95% CI	P-Value
NWSS	3.08	0.792–12.0	0.105
OSI	4.47E-05	1.47e-11–135	0.188
Step 3			
Parameter	OR	95% CI	P-Value
NWSS	4.81	1.37–16.9	0.0141

CI indicates confidence interval; NWSS, normalized wall shear stress; OR, odds ratio; OSI, oscillatory shear index; and RRT, relative residence time.

## Discussion

The elderly patients may have an increased incidence of atherosclerotic changes of cerebral aneurysms and its parent arteries. The risk of treating aneurysms with atherosclerosis by surgical procedures includes ischemic events.[12][13][14] In addition, the atherosclerosis affects the elasticity of the arterial wall and aneurysm wall, and the resultant partially rigid arterial wall may hamper temporary clipping of parent artery, that is, causing incomplete obliteration of the clip blades. Therefore, prediction of the HR lesions on cerebral aneurysms and parent arteries before surgery would contribute to avoiding intraoperative risks by facilitating preoperative simulation such as clipping work.

To the best of our knowledge, this is the first report evaluating several hemodynamic parameters that have possible association with HR lesions simultaneously. The relationship between intraoperative findings of atherosclerosis on cerebral aneurysms and local hemodynamics has not been fully examined. In previous reports, prolonged RRT of cerebral aneurysms had a possible association with atherosclerosis.[9] In addition, high OSI was observed around atherosclerotic changes on cerebral aneurysms and parent arteries.[10]

RRT is a hemodynamic parameter to detect stagnation flow and is related with atherosclerosis of the other vascular territories.[4][6][15] OSI quantified the fluctuation of WSS vector, and high OSI corresponded with the atherosclerosis of carotid artery,[7] aortic arch,[15] and abdominal aorta.[5] On the other hand, CFD can evaluate other kinds of fluctuation of WSS vector besides OSI. AFI was originally developed to evaluate aneurysm initiation and to detect the flow stagnation by comparing the angle of WSS vector at midsystolic deceleration.[11] Some previous reports showed that atherosclerosis had a correlation with low WSS or high OSI.[5][7][16][17] In this study, the distribution of high OSI was similar to that of low AFI, and the both hemodynamic characteristics were commonly observed at low WSS or NWSS zones. In addition, low NWSS, high OSI, prolonged RRT and low AFI corresponded with the HR or arteriosclerotic lesions well. These findings indicate that HR lesions could be caused by not only low WSS but also high fluctuation of the WSS vector. WSS is changing during one cardiac cycle, and therefore momently zero of the WSS magnitude or the very small time-averaged WSS does not mean zero or nearly zero WSS vector through one cardiac cycle. That is, “zero (or near zero) of WSS vector” or “oscillating very small WSS vector” is not equal to “oscillating zero”. We think that small WSS vector and high oscillation mean flow stagnation at the regions or regions near the flow separation, being a trigger of functional regulation of endothelial cells. Actually, these physiological reactions were demonstrated to occur under low WSS with around 1 Pa.[17]

Our findings suggest a possible relation between hemodynamic parameters indicating oscillation of WSS vector and atherogenesis on cerebral aneurysm wall or vessel wall, although there is no evidence of a causal relation. However, WSS and WSS-related hemodynamic parameters including OSI have been reported to be related to arteriosclerosis on carotid artery, aortic arch, and abdominal aorta,[5][7][15] and biomechanical links between disturbed blood flow and atherosclerosis are well established.[17][18] Previous studies demonstrated that endothelial responses to hemodynamic factors played an important role in atherogenic transformation.[7][16][19] Malek et al [17] also reported biological responses of the endothelium to WSS by using aortic endothelial cells of rats. In particular, reduction of WSS has been associated with endothelial proliferation: endothelial cells are damaged by blood flow with low WSS and high oscillation of WSS vector, which activates focal inflammatory process, resulting in atherosclerosis and proliferation of endothelial cells.[7][16][17][18][19][20] Although these studies were implemented using normal endothelial cells of animals, it may be reasonable to consider that these kinds of physiological reactions occur in the HR of cerebral aneurysm walls.

This article has several limitations. First, comparison of intraoperative view and virtual view was conducted after surgery. In a strict sense, thus, this study did not perform preoperative prediction of HR lesions. Second, all CFD simulations employed the same boundary conditions, although it is unknown if the boundary condition should be set for each location and size of aneurysms. Third, the determination of HR lesions, red walls, and qualification points was subjective, although 2 experienced neurosurgeons blinded to the results of CFD analyses determined them by consensus to reduce selection bias. Fourth, the qualification points were set at the center of the lesions, because the size of the lesions were various (ranging from only a small part to most of the aneurysm wall), and therefore it was difficult to determine the standard size of region of interest. However, there were often differences between the outer shape and the vessel lumen of cerebral aneurysms because of the atherosclerotic HR of the aneurysm wall (for example, Fig 3a): the geometry model of cerebral aneurysm used for CFD analysis reflects the shape of the vessel lumen, whereas the outer shape of cerebral aneurysm is observed in intraoperative images. Therefore, it might not always be possible to select exactly the same position on both the geometry model image and the intraoperative image. Thus, further studies are needed to determine whether preoperative prediction of HR lesions on aneurysmal walls is reliably possible or not.

## Conclusions

In addition to low WSS, fluctuation of WSS vectors, OSI, may be a key hemodynamic parameter to predict HR lesions on cerebral aneurysms. These results suggest that CFD could predict the presence of HR lesions on aneurysm walls before surgical treatment.

## Supporting information

S1 File Tables 1, 2, 3 and 4 information file. (XLSX)
